# Unraveling the role of ChREBP in lung adenocarcinoma: Expression, regulatory networks, and potential functional impact

**DOI:** 10.1371/journal.pone.0347907

**Published:** 2026-04-30

**Authors:** Athisake Ruangpracha, Chanachai Sae-Lee, Pitaksin Chitta, Harald Grove, Pranisa Jamjuntra, Warisa Amornrit, Prapat Suriyaphol, Chanitra Thuwajit, Naravat Poungvarin

**Affiliations:** 1 Doctor of Philosophy Program in Medical Biochemistry and Molecular Biology (International Program), Department of Biochemistry, Faculty of Medicine Siriraj Hospital, Mahidol University, Bangkok, Thailand; 2 Clinical Molecular Pathology Laboratory, Department of Clinical Pathology, Faculty of Medicine Siriraj Hospital, Mahidol University, Bangkok, Thailand; 3 Research Department, Faculty of Medicine Siriraj Hospital, Mahidol University, Bangkok, Thailand; 4 Department of Animal and Aquacultural Sciences, Faculty of Biosciences, Norwegian University of Life Science, Ås, Norway; 5 Department of Immunology, Faculty of Medicine Siriraj Hospital, Mahidol University, Bangkok, Thailand; Sichuan University, CHINA

## Abstract

Carbohydrate Response Element Binding Protein (ChREBP) is a transcription factor known to regulate glucose metabolism and other metabolic processes in various tissues, but its role in lung adenocarcinoma (LUAD) remains poorly understood. In this study, we investigated ChREBP expression and its role in regulating gene expression in LUAD cell lines. Using RT-qPCR, we assessed the expression of ChREBP-α and ChREBP-β isoforms in NCI-H1975, NCI-H1650, and NCI-H2228 LUAD cell lines. The NCI-H1975 cells exhibited the highest levels of both ChREBP isoforms, with a particularly pronounced expression of ChREBP-β. To explore the regulatory role of ChREBP, we generated NCI-H1975 cells with inducible expression of a dominant-negative mutant of human ChREBP (dnChREBP). Overexpression of dnChREBP led to a significant reduction in colony formation and impaired cell migration. Transcriptome analysis revealed 57 upregulated genes and 593 downregulated genes in dnChREBP-expressing cells compared to control cells. Functional annotation and gene set enrichment analysis revealed that the enriched genes were associated with cancer-related processes, including cell proliferation and epithelial-to-mesenchymal transition (EMT). Gene network analysis highlighted 17 downregulated hub genes, with 8 of these genes being associated with EMT. Interestingly, ChREBP and its transcriptionally regulated genes, including 4 top downregulated genes, 5 top upregulated genes, and 5 hub genes identified in NCI-H1975 cells overexpressing dnChREBP, showed significant prognostic value, as their expression levels correlated with overall survival in LUAD patients. Our findings suggest that ChREBP regulates distinct transcriptional programs in LUAD cells and ChREBP and its regulatory network may play a potential role in LUAD progression and patient outcomes.

## Introduction

Carbohydrate response element binding protein (ChREBP), also known as *MLXIPL*, is a multidomain transcription factor that plays a central role in regulating the transcription of genes involved in glucose and lipid metabolism [1,2]. ChREBP is expressed across various tissues, with the highest levels observed in the liver [3]. The ChREBP-α is the full-length isoform of ChREBP which is transcriptionally inactive under low glucose condition. High glucose levels relieve this inhibition, leading to the translocation of ChREBP-α into the nucleus, where it becomes active. [4–6]. On the other hand, ChREBP-β, a shorter isoform lacking most of the N-terminal low glucose inhibitory domain, is constitutively active, independent of glucose levels [7].

ChREBP forms a heterodimer with Max-like protein X (MLX) and binds to carbohydrate response elements (ChoRE) in the promoters of target genes [8,9]. Through this mechanism, ChREBP regulates key metabolic pathways by controlling the expression of genes such as fatty acid synthase (*FASN*), MID1 Interacting Protein 1 (*MID1IP1*), acetyl-CoA carboxylase alpha (*ACACA*), ELOVL fatty acid elongase 6 (*ELOVL6*), stearoyl CoA desaturase 1 (*SCD1*), glycerol-3-phosphate dehydrogenase 1 (*GPD1*), diacylglycerol O-acyltransferase 2 (*DGAT2*), fructokinase (*KHK*), peroxisome proliferator activated receptor alpha (*PPARA*), Krüppel-like factor-10 (*KLF10*), solute carrier family 6 (neurotransmitter transporter, glycine), member 9 (*SLC6A9*), pyruvate dehydrogenase kinase isozyme 2 (*PDK2*), and tribbles pseudokinase 3 (*TRIB3*) [10–12].

Metabolic pathways are highly active in human cancers [13–16]. Metabolic reprogramming has emerged as a key driver of carcinogenesis and cancer progression, as cancer cells exploit these pathways to facilitate proliferation, survival, migration, and invasion [17,18]. Cancers exhibit a pronounced activation of glycolytic pathways, fulfilling the high-energy and biosynthetic demands of rapidly dividing cells.

Several evidences suggested that ChREBP plays significant roles not only in human health, but also in various diseases, including metabolic syndrome and cancers [19–21]. High ChREBP expression has been observed in several types of solid cancers, such as liver, colorectal, breast, and prostate cancers, and it has been linked to the progression of these malignancies [22–26].

Information regarding ChREBP expression in lung adenocarcinoma (LUAD), one of the most prevalent cancers globally, remains limited [27]. One study has reported that Snail1, a transcription factor mediating TGFβ1-induced EMT in the A549 LUAD cell line, suppresses both *FASN* and ChREBP. Much remains to be uncovered about ChREBP expression and function in LUAD [27]. Therefore, the objective of this study is to evaluate ChREBP expression across different LUAD cell lines and to explore ChREBP-regulated genes and pathways, aiming to provide insights into the potential roles of ChREBP in LUAD cells.

## Materials and methods

### Cell culture

The NCI-H1975 (ATCC CRL-5908), NCI-H1650 (ATCC CRL-5883), NCI-H2228 (ATCC CRL-5935), and HepG2 (HB-8065) cell lines were obtained from the American Type Culture Collection (ATCC, USA). No human participants or animals were involved; therefore, ethical approval was not required. LUAD cell lines were cultured in Roswell Park Memorial Institute Medium 1640 (RPMI 1640) (ATCC, USA) supplemented with 10% fetal bovine serum (FBS) (Gibco, USA). The HepG2 cell line was maintained in Eagle’s Minimal Essential Medium (ATCC, USA) with 10% FBS (Gibco, USA). Human embryonic kidney 293T (HEK293T) cells were cultured in Dulbecco#39;s Modified Eagle Medium (ATCC, USA) supplemented with 10% FBS (Gibco, USA). All cell cultures were incubated at 37°C in a humidified atmosphere containing 5% CO2.

### Lentiviral vector construction

We utilized pTRIPZ self-inactivating lentiviral vector (Open Biosystems, USA) for Tet-on inducible expression of the transgene. The lentiviral vector construct for the inducible nuclear form of enhanced yellow fluorescent protein (eYFP) was previously described [11]. N-terminal Myc-tagged dnChREBP was generated by amplifying a fragment of the human *MLXIPL* gene from HepG2 cDNA and inserting it into the AgeI and MluI sites of the pTRIPZ vector. The primer sequences used were as follows: Myc-dnChREBP forward primer: 5′-AATTaccggtGCCACCATGGAGCAGAAACTCATCTCTGAAGAGGATCTGGCCCCTTCCAGGCCCCT-3′ and Myc-dnChREBP reverse primer: 5′-AATTacgcgtCTATAAAGGTTTGCCAAGGGT-3′. All constructs were verified by Sanger sequencing.

### Generation of inducible cell lines

We transfected inducible lentiviral vector construct, along with psPAX2 and pMD2.G plasmids into HEK293T cells using calcium phosphate method. The resulting lentiviral vectors were transduced into NCI-H1975 cells in the presence of 8 μg/mL polybrene (Sigma, USA). Successfully transduced cells were selected using 2 μg/mL puromycin (Calbiochem, Germany).

Inducible cells were seeded at a density of 500,000 cells per 35-mm culture dish. At 12 hours, we incubated the cells with cultured medium containing 5.5 mM D-glucose (Gibco, USA) and 2 μg/mL doxycycline (Bio Basic Inc, Canada). Following a 24-hour incubation, the medium was changed to one containing 25 mM D-glucose and 2 μg/mL doxycycline. Cells were harvested after 48 hours for further analysis.

### RNA extraction

Total RNA was extracted from cell lines using the Total RNA Mini Kit for Blood/Culture cells (Geneaid, Taiwan) following the manufacturer#39;s protocol. RNA concentration and quality were assessed using a FLUOstar Omega instrument with an LVis Plate (BMG Labtech, Germany).

### Reverse Transcription and Quantitative PCR (RT-qPCR)

Reverse transcription was performed to synthesize cDNA from the extracted RNA using Omniscript RT kit (Qiagen, Germany). Quantitative PCR assays were conducted to measure the expression levels of genes. The expression stability of candidate HKGs was evaluated using the geNorm [28]. The three most stable HKGs were selected for normalization of gene expression levels. The sequences of the primers used in this study are provided in S1 Table.

### Transcriptome sequencing and bioinformatic analysis

RNA sequencing was performed by Macrogen using the Illumina NextSeq500 platform following standard protocols recommended by Illumina. All bioinformatics analyses were conducted on the Galaxy platform [29]. Raw sequencing reads were quality-filtered and trimmed using Trimmomatic [30], followed by quality assessment with FASTQC. Cleaned reads were aligned to the Homo sapiens genome (hg38) using HISAT2 with default settings [31]. Transcript quantification was performed with FeatureCounts [32], and differential expression analysis was carried out using DESeq2 [33]. Differentially expressed genes (DEGs) were identified based on a threshold of |log2 FC| ≥ 2 and an adjusted p-value < 0.05 (Benjamini-Hochberg correction). Gene Ontology (GO) enrichment and MSigDB hallmark analyses [34] were performed using Enrichr [35–37]. Protein-protein association networks were constructed using STRING [38]. The ggplot2 package was used to generate volcano plot.

### Colony formation assay

Cells were seeded at 2,000 cells/well in 24-well plates and cultured in complete growth medium for 7 days. Colonies were fixed with 4% paraformaldehyde in PBS and stained with 0.5% crystal violet (Sigma-aldrich, Germany) for visualization. Stained colonies were counted using ImageJ [39].

### Cell migration analysis

Cells were seeded into 24-well transwell insert plates at a density of 20,000 cells per well. After 12 hours, the cells were incubated with culture medium containing 5.5 mM D-glucose (Gibco, USA) and 2 μg/mL doxycycline (Bio Basic Inc, Canada). Following a 24-hour incubation period, the medium was replaced with fresh medium containing 25 mM D-glucose and 2 μg/mL doxycycline for an additional 48 hours. Cell migration was assessed at 72 hours after seeding (12 hours post-attachment). The inserts were fixed with methanol for 15 minutes and stained with 0.5% crystal violet for 30 minutes. Migrated cells were then counted under a microscope.

### Statistical analysis

Statistical comparisons between two groups were made using the Student#39;s t-test. Data are presented as mean ± standard deviation (SD), with significance determined at a p-value or adjusted p-value < 0.05.

### Survival analysis

Survival analysis was performed using the KMplot database [40]. Patients were divided into high and low expression groups using the auto-select best cutoff option, which computes all possible cutoff values between the lower and upper quartiles and selects the threshold that provides the most significant separation between the two groups.

## Results

### Both ChREBP-α and ChREBP-β transcripts are expressed in human LUAD cells

ChREBP is prominently expressed in human hepatocytes and its expression has been demonstrated in liver cancer cells, including the HepG2 hepatocellular carcinoma cell line [12,23,41]. In the context of human LUAD cells, ChREBP expression has only been studied in the A549 cell line [42]. Notably, the PCR primers used in that study targeted only the ChREBP-α isoform, leaving the expression of ChREBP-β unexamined. To date, no data has been published on the expression of the ChREBP-β isoform in human LUAD cells.

**Fig 1:**
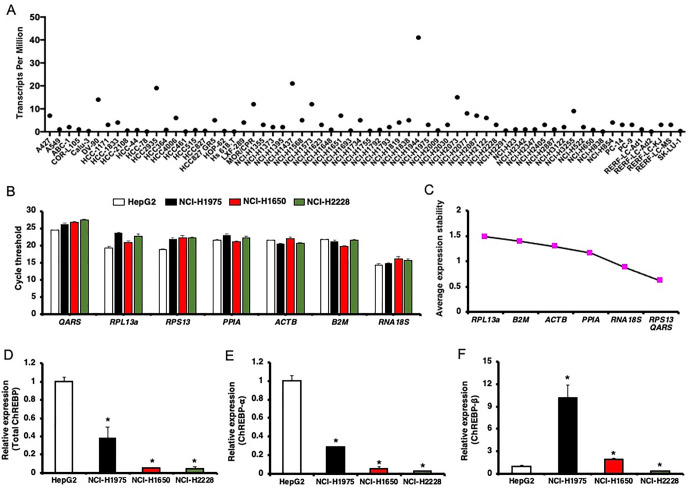
ChREBP expression in human LUAD cell lines. (A) Expression levels of ChREBP (*MLXIPL*) in 66 human LUAD cell lines, derived from RNA-seq datasets available in the EMBL-EBI Expression Atlas, are presented in transcripts per million (TPM). (B-F) Expression of ChREBP in NCI-H1975, NCI-H1650, NCI-H2228 and HepG2 cells assessed using RT-qPCR assays. (B) Expression level of seven candidate HKGs. Cycle threshold (Ct) values are displayed. (C) Average expression stability values of seven HKGs determined by geNorm are shown. Expression levels of total ChREBP (D), ChREBP-α (E), and ChREBP-β (F) were normalized against the three most stable HKGs, *RPS13, QARS,* and *RNA18S*. Data represents the results of three independent experiments. Asterisks (*) indicate a significance level of *p* < 0.05 compared to the expression levels in the HepG2 cell line.

In our study, we initially assessed ChREBP expression across various cell lines using RNA-seq data retrieved from the Expression Atlas database (http://www.ebi.ac.uk/gxa) [43]. Our analysis identified varying levels of ChREBP expression among 66 human LUAD cell lines (Fig 1A). Ten cell lines exhibited ChREBP expression below the designated cut-off (transcripts per million, TPM < 0.5). These cells are HCC-78, HCC461, Hs 618.T, NCI-H838, RERF-LC-Ad2, Calu-3, HOP-62, SK-LU-1, HCC827, and NCI-H1755. The majority of LUAD cell lines expressed low levels of ChREBP transcripts (0.5 ≤ TPM < 10), including A549, NCI-H1975, and NCI-H2228. Seven cell lines showed moderate ChREBP expression (11 ≤ TPM < 1000). These cells are MOR/CPR, NCI-H1573, DV-90, NCI-H2073, HCC2935, NCI-H1437, and NCI-H1944. Isoform-specific ChREBP expression data were not available in the EMBL-EBI Expression Atlas.

To gain more insight into ChREBP expression in human LUAD cell lines, we performed RT-qPCR to investigate expression of total ChREBP, ChREBP-α, and ChREBP-β expression in NCI-H1975, NCI-H1650, and NCI-H2228 cells, comparing the results to those from the HepG2 cell line. Since the selection of appropriate housekeeping genes (HKGs) is crucial for accurate gene expression comparison across different cell types, we therefore evaluated the expression of seven commonly used HKGs to identify the most stable HKGs. Our analysis revealed *RNA18S* (average CT values ranging from 14.36 to 16.05) as the highest expressed gene and *QARS* (average CT values ranging from 24.37 to 27.50) as the lowest expressed gene (Fig 1B). geNorm analysis identified *RPS13* (CT values ranging from 18.78 to 22.18), *QARS* (CT values ranging from 24.37 to 27.50), and *RNA18S* (CT values ranging from 14.36 to 16.05) as the three most stable HKGs, whereas *RPL13A* (CT values ranging from 19.33 to 23.57) was the least stable (Fig 1B and 1C). Interestingly, *ACTB*, a commonly used HKG, was not identified as an ideal HKG in our analysis.

Normalization using the three most stable HKGs revealed that total ChREBP expression was highest in NCI-H1975 cells among the LUAD cell lines tested (Fig 1D). The NCI-H2228 cells exhibited the lowest total ChREBP expression. Levels of total ChREBP expression in NCI-H1975, NCI-H1650, and NCI-H2228 cells were 2.7-fold, 17.2-fold, and 23.9-fold lower than in HepG2 cells, respectively. Isoform-specific RT-qPCR analysis indicated that ChREBP-α expression mirrored the total ChREBP expression pattern in these LUAD cells (Fig 1E). ChREBP-α expression levels in NCI-H1975, NCI-H1650, and NCI-H2228 cells were 3.5-fold, 18.0-fold, and 30.2-fold lower than in HepG2 cells, respectively. Interestingly, ChREBP-β expression followed a distinct pattern. NCI-H1975 cells exhibited the highest ChREBP-β expression, with levels 10.1-fold higher than in HepG2 cells (Fig 1F). In NCI-H1650 cells, ChREBP-β expression was 1.9-fold higher than in HepG2, whereas NCI-H2228 cells exhibited a 3.7-fold lower expression level compared to HepG2 cells.

In summary, the majority of human LUAD cell lines express ChREBP transcripts, although the levels of expression vary between lines. Not only ChREBP-α, but also ChREBP-β isoform, were detected in the LUAD cell lines NCI-H1975, NCI-H1650, and NCI-H2228. Notably, NCI-H1975 cells demonstrated the most pronounced expression of ChREBP-β among the cell lines examined in this study. Since ChREBP is a transcription factor capable of regulating the expression of numerous genes across various cell types, we hypothesized that it may also play an important regulatory role in the molecular pathways of LUAD cells.

### Overexpression of dnChREBP reduces colony formation in LUAD cells

**Fig 2:**
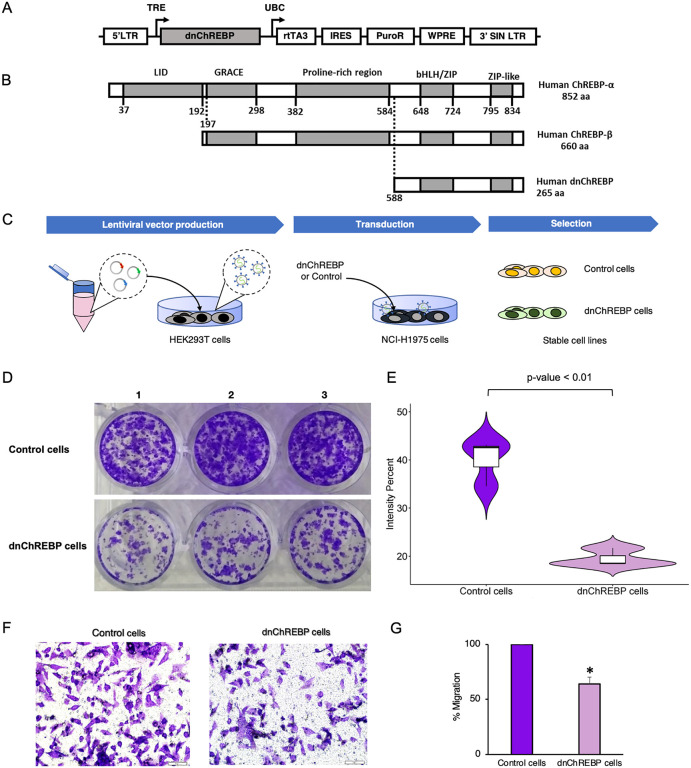
Impact of dominant negative ChREBP (dnChREBP) overexpression on colony formation ability and migration in NCI-H1975 LUAD cells. (A) Schematic representation of doxycycline-inducible vectors used for overexpression of dnChREBP. LTR, long terminal repeat; TRE, tetracycline-responsive promoter element; UBC, human ubiquitin C promoter; rtTA3, reverse tetracycline-transactivator 3; IRES, internal ribosomal entry site; PuroR, puromycin resistance gene; WPRE, Woodchuck hepatitis posttranscriptional regulatory element; SIN LTR, self-inactivating long terminal repeat. (B) Amino acid alignment of human ChREBP-α and ChREBP-β. bHLH, basic Helix-Loop-Helix-Leucine; ZIP, leucine zipper. (C) Outline of the process used to generate dnChREBP-overexpressing NCI-H1975 cells. (D-E) Colony formation assay. Macroscopic visualization of the crystal violet-stained colonies is shown (D). Violin plot showing the intensity distribution of stained colonies formed in dnChREBP-overexpressing NCI-H1975 cells compared to control cells (E). A significant difference (p-value < 0.01) is indicated. (F-G) Transwell migration assay. Representative images of migrated control and dnChREBP cells at 20x magnification is shown (F). The bar graph shows the percentage of migrated cells, with data representing the mean ± SD from three independent experiments (G).

To better understand the role of ChREBP in LUAD, we developed NCI-H1975 cell lines with inducible expression of either a dominant-negative form of ChREBP (dnChREBP) or a control (eYFP) using self-inactivating lentiviral vectors (Fig 2A and C). The dnChREBP is a human ChREBP variant lacking the transactivation domain in comparison to the full-length ChREBP (Fig 2B), leading to the functional inactivation of ChREBP.

We observed slower growth in dnChREBP-overexpressing cells compared to control cells. To further investigate this phenomenon, we conducted a colony formation assay and found that dnChREBP overexpression significantly reduced colony formation in NCI-H1975 LUAD cells, with the intensity of colony formation decreasing to 48.8% compared control cells (Fig 2D and E). This impairment in proliferative capacity occurred despite no significant differences in short-term cell viability (MTS assay) or in the activation states of key growth signaling pathways (pERK and pAKT levels) at 72 hours (S1 Fig). We noted that the dnCHREBP-overexpressing cells generally maintained normal cellular morphology comparable to that of control cells.

### Transcriptome sequencing reveals ChREBP-regulated genes in LUAD cells

**Fig 3:**
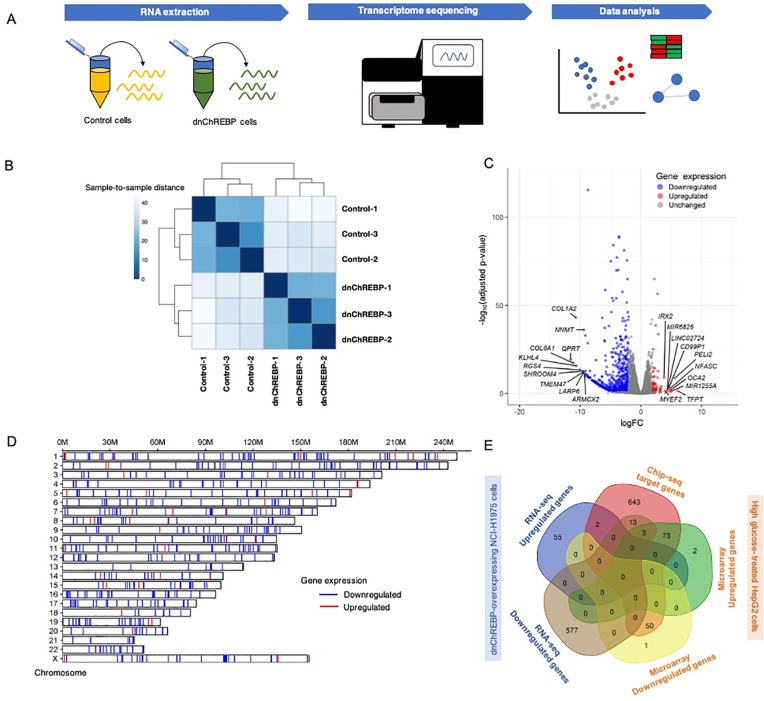
Transcriptome analysis of NCI-H1975 LUAD cells overexpressing dnChREBP. (A) Brief diagram outlining the key steps of RNA sequencing analysis. (B) Heatmap displaying sample-to-sample distances calculated from the variance-stabilizing transformation of count data for overall gene expression. (C) Volcano plot illustrating differentially expressed genes (DEGs); red dots represent upregulated genes, blue dots indicate downregulated genes, and grey dots denote unchanged genes (adjusted p-value < 0.05). (D) Chromosomal localization of DEGs. The DEGs are plotted at their chromosomal positions.

We performed RNA sequencing (RNA-seq) to assess the impact of dnChREBP expression in regulating gene expression in NCI-H1975 cells (Fig 3A). The RNA-seq data derived from three biological replicates each of dnChREBP cells and control cells revealed a clear distinction in gene expression profiles between the two groups (Fig 3B). Our analysis identified 57 upregulated genes and 593 downregulated genes that were differentially expressed (|log2 FC| ≥ 2 and adjusted p-value < 0.05) (Fig 3C and S2 Table). The chromosomal locations of these differentially expressed genes are distributed across all chromosomes except the Y chromosome (Fig 3D), which is consistent with the female origin of the NCI-H1975 cell line used in this study. The top 10 downregulated genes included quinolinate phosphoribosyltransferase (*QPRT*), collagen type VIII alpha 1 chain (*COL8A1*), collagen type I alpha 2 chain (*COL1A2*), kelch like family member 4 (*KLHL4*), regulator of G protein signalling 4 (*RGS4*), shroom family member 4 (*SHROOM4*), transmembrane protein 47 (*TMEM47*), la ribonucleoprotein 6, translational regulator (*LARP6*), nicotinamide N-methyltransferase (*NNMT*), and armadillo repeat containing x-linked 2 (*ARMCX2*); while the top 10 upregulated genes included TCF3 fusion partner (*TFPT*), pellino E3 ubiquitin protein ligase family member 2 (*PELI2*), family with sequence similarity 149 member A (*FAM149A*), microRNA 1255A (*MIR1255A*), dickkopf wnt signaling pathway inhibitor 4 (*DKK4*), OCA2 melanosomal transmembrane protein (*OCA2*), neurofascin (*NFASC*), CD99 molecule pseudogene 1 (*CD99P1*), long intergenic non-protein coding RNA 2724 (*LINC02724*), and microRNA 6826 (*MIR6826*).

### Functional annotation and network analysis uncover potential ChREBP-regulated pathways and genes in LUAD cells

**Fig 4:**
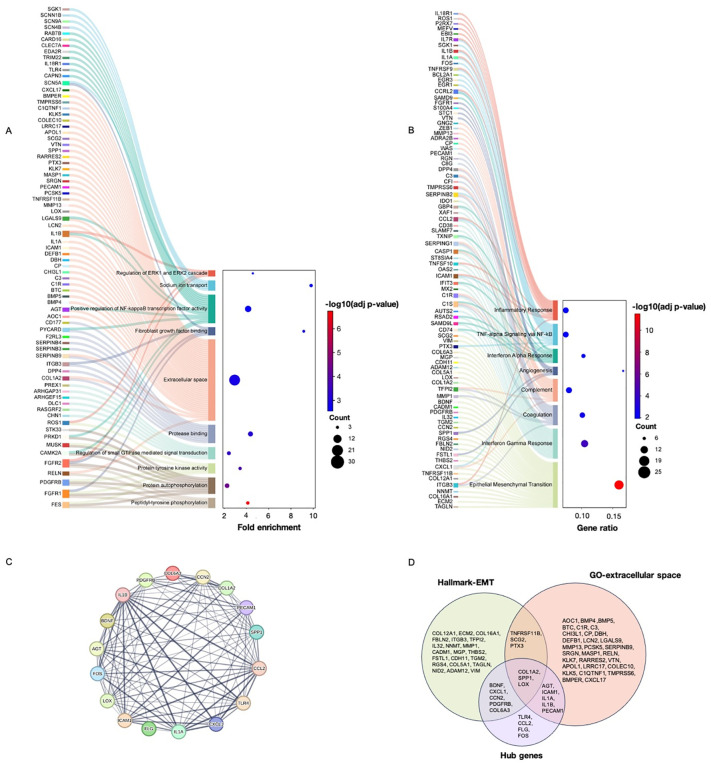
Enrichment analysis of DEGs associated with dnChREBP overexpression in NCI-H1975 cells. (A) Gene ontology (GO) analysis displaying the enriched biological processes related to DEGs, indicating fold enrichment and significance levels (−log10 adjusted p-value). (B) Hallmark gene sets highlighting significant pathways associated with the expression changes. The gene ratio and adjusted p-values are shown. (C) Interaction network of hub genes, illustrating their interconnections and potential regulatory relationships. (D) Venn diagram illustrating the overlap of genes associated with the EMT Hallmark, the extracellular space GO term, and the identified hub genes.

**Fig 5:**
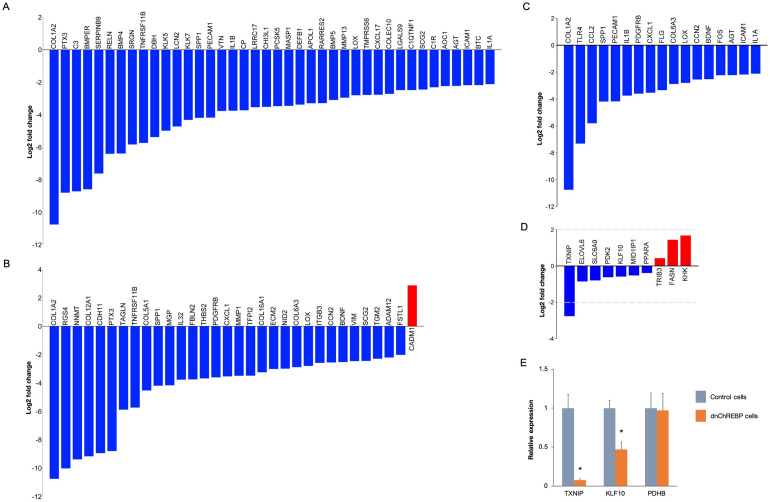
Alteration of expression levels of selected genes in dnChREBP-overexpressing NCI-H1975 cells. (A-D) RNA-seq results. Bar graphs depict the Log2 FC in genes enriched in the extracellular space GO term (A), genes enriched in the Hallmark EMT gene set (B), hub genes (C), and ChREBP target genes (D). (E) Gene expression analysis using RT-qPCR was conducted on selected genes to validate the RNA-seq findings. Gene expression levels were normalized against the three most stable HKGs, *RPL13a*, *ACTB*, and *B2M*. Asterisks (*) denote statistically significant differences (*p* < 0.05) compared to the expression levels in control cells.

To explore the functional significance of DEGs associated with dnChREBP in NCI-H1975 LUAD cells, we conducted a comprehensive functional annotation. Gene Ontology (GO) analysis highlighted various significantly enriched pathways (adjusted p-value <0.05) relevant to cancer, including transcription coregulator binding, regulation of small GTPase mediated signal transduction, regulation of ERK1 and ERK2 cascade, protein tyrosine kinase activity, protein autophosphorylation, positive regulation of NF-κB transcription factor activity, positive regulation of cell population proliferation, peptidyl-tyrosine phosphorylation FGF binding and extracellular space (Fig 4A and S3 Table). Among these, the extracellular space GO term contained the highest number of enriched genes (40 genes) (Fig 4D and 5A). Additionally, hallmark gene set enrichment analysis indicated involvement in epithelial-to-mesenchymal transition (EMT), interferon gamma response, coagulation, complement, angiogenesis, interferon alpha response, TNF-alpha signalling via NF-kB and inflammatory response (Fig 4B and S3 Table). Among these, the EMT gene set exhibited the highest number of enriched genes (24 genes) (Fig 4D and 5B). To provide experimental support for the EMT-related gene expression changes, we performed transwell migration assays, which revealed significantly impaired migratory capacity (36% reduction) in dnChREBP-overexpressing cells compared to controls (Fig 2F-G).

We also constructed an interaction network to identify potential hub genes influenced by dnChREBP expression. This analysis revealed 17 key genes, all of which were downregulated in dnChREBP-overexpressing cells (Fig 4C-D and 5C). These included angiotensinogen (*AGT*), brain derived neurotrophic Factor (*BDNF*), C-C motif chemokine ligand 2 (*CCL2*), cellular communication network factor 2 (*CCN2*), *COL1A2*, collagen type VI alpha 3 chain (*COL6A3*), C-X-C motif chemokine ligand 1 (*CXCL1*), filaggrin (*FLG*), fos proto-oncogene, AP-1 transcription factor subunit (*FOS*), intercellular adhesion molecule 1 (*ICAM1*), interleukin 1 alpha (*IL1A*), interleukin 1 beta (*IL1B*), lysyl oxidase (*LOX*), platelet derived growth factor receptor beta (*PDGFRB*), platelet and endothelial cell adhesion molecule 1 (*PECAM1*), secreted phosphoprotein (*SPP1*), and toll like receptor 4 (*TLR4*). The top three most downregulated hub genes are *COL1A2*, *TLR4*, and *CCL2*, with log2 FC of −10.75, −7.31 and −5.80, respectively.

Thirty-two genes in the EMT hallmark gene set exhibited differential expression in dnChREBP-overexpressing cells (Fig 4D and 5B). Among these, only cell adhesion molecule 1 (*CADM1*) was upregulated. The downregulated EMT-related genes included ADAM metallopeptidase domain 12, cadherin 11, collagen type XII alpha 1 chain, collagen type XVI alpha 1 chain, collagen type V alpha 1 chain (*COL5A1*), extracellular matrix protein 2, fibulin 2 (*FBLN2*), follistatin like 1 (*FSTL1*), interleukin 32 (*IL32*), integrin subunit beta 3 (*ITGB3*), matrix Gla protein, matrix metallopeptidase 1 (*MMP1*), nidogen 2*, NNMT,* pentraxin 3 (*PTX3*)*, RGS4,* secretogranin II, transgelin (*TAGLN*), tissue factor pathway inhibitor 2, transglutaminase 2, thrombospondin 2, TNF receptor superfamily member 11b, and vimentin (*VIM*).

We additionally investigated the expression of known human ChREBP target genes [8,12,44] in dnChREBP-overexpressing cells (Fig 5D). Notably, thioredoxin interacting protein (TXNIP) was the only target gene analyzed that displayed a significant decrease with a |log2 FC| ≥ 2. Several ChREBP target genes, including *ELOVL6, SLC6A9, PDK2, KLF10, MID1IP1, PPARA*, exhibited significant downregulation (adjusted p-value <0.05), although with |log2 FC| values below 2. Similarly, tribbles pseudokinase 3 (*TRIB3*), *FASN,* and ketohexokinase (*KHK*), exhibited significant upregulation (adjusted p-value <0.05), though their |log2 FC| values were also below 2. The expression of pyruvate kinase L/R (*PKLR*)*, INHBE, ACACA, GPD1,* and *DGAT2* is not significantly different.

### Survival analysis of ChREBP and Associated Genes in LUAD Patients

**Fig 6:**
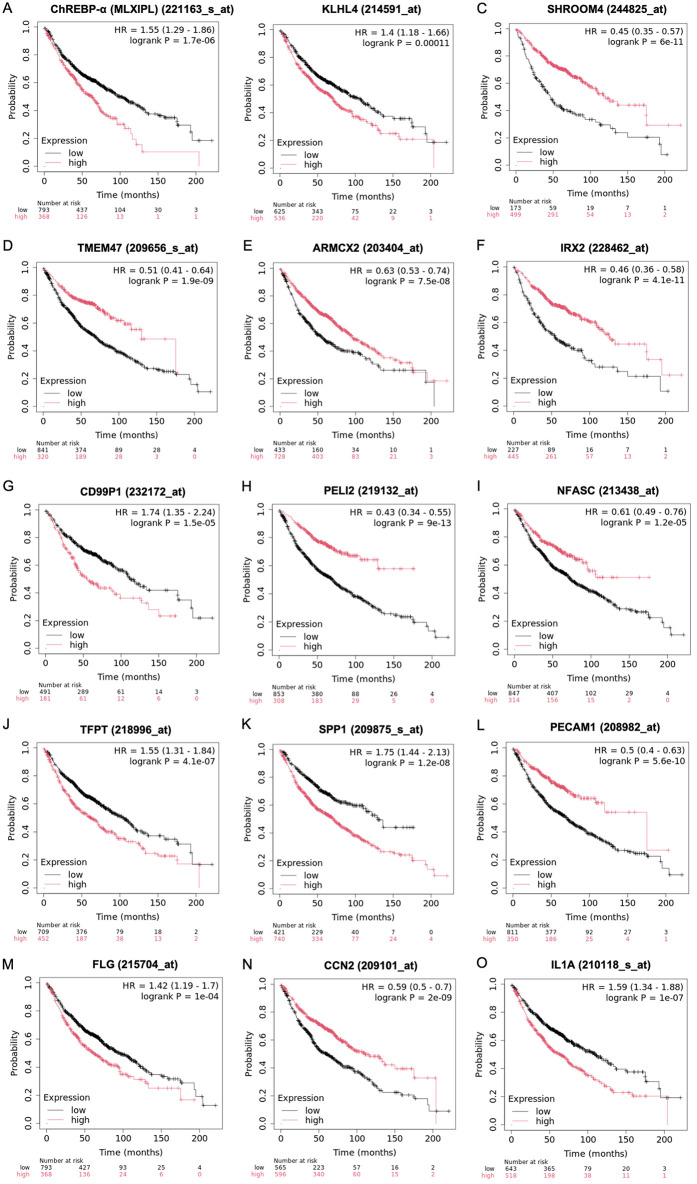
Prognostic significance of ChREBP and its associated genes in LUAD. Kaplan–Meier plot demonstrates the median overall survival (OS) of LUAD patients. Only genes with statistical significance (*p* < 0.05) and a false discovery rate (FDR) of 1% are shown. (A) ChREBP-α (*MLXIPL*). (B-E) Top 10 downregulated genes by dnChREBP in NCI-H1975 cells: *KLHL4* (B), *SHROOM4* (C), *TMEM47* D) and *ARMCX2B* (E). (F-J) Top 10 upregulated genes by dnChREBP in NCI-H1975 cells: *IRX2* (F), *CD99P1* (G), *PELI2* (H), *NFASC* (I), and *TFPT* (J). (K-O) Hub genes: *SPP1* (K), *PECAM1* (L), *FLG* (M), *CCN2* (N), *IL1A* (O).

We utilized the Kaplan–Meier plotter to demonstrated the median overall survival (OS) of LUAD patients, stratified into low-expression and high-expression cohorts based on the expression levels of each gene [40]. Our analysis revealed significant associations between overall survival and expression levels of ChREBP (*MLXIPL*) (Fig 6A). Patients with low ChREBP expression had a median OS of 99 months, while those with high ChREBP expression had a significantly shorter median OS of 62 months.

In addition, we analyzed the median OS of LUAD patients based on the expression levels of genes from the top 10 downregulated genes, top 10 upregulated genes, and 17 hub genes identified in NCI-H1975 cells overexpressing dnChREBP. Significant differences in median OS were observed for: *KLHL4*, *SHROOM4*, *TMEM47*, and *ARMCX2* (top 10 downregulated genes) (Fig 6B-E); *IRX2*, *CD99P1*, *PELI2*, *NFASC*, and *TFPT* (top 10 upregulated genes) (Fig 6F-J); and *SPP1*, *PECAM1*, *FLG*, *CCN2*, and *IL1A* (17 hub genes) (Fig 6K-O). To assess the statistical robustness of these findings, we applied Benjamini–Hochberg false discovery rate (FDR) correction across all 37 tested genes (S4 Table). All 14 identified genes remained statistically significant after correction (FDR-adjusted p < 0.05).

These findings suggest that the expression levels of ChREBP and its associated genes are strongly correlated with survival outcomes in LUAD patients, underscoring their potential as prognostic markers in LUAD.

## Discussion

Accumulating evidence supports the crucial role of ChREBP in cancer development, growth, and progression [26,45,46]. Existing studies predominantly focus on total ChREBP expression, lacking detailed insights into individual ChREBP isoform expression. Progression of breast and colon cancers is associated with an increase in total ChREBP mRNA and protein levels in clinical samples [24,26]. Similarly, upregulation of total ChREBP transcripts is detected in human metastatic prostate cancer tissue [47].

Current evidence on the role of ChREBP in lung cancer is extremely limited. To date, ChREBP-α expression in LUAD cells other than the A549 cell line, ChREBP-β expression, and the functional role of ChREBP remain unexplored. Our analysis of publicly available RNA-seq datasets demonstrates that ChREBP is expressed in the majority of LUAD cell lines. While all three LUAD lines in our study exhibited lower total ChREBP levels compared to HepG2 cells, NCI-H1975 and NCI-H1650 displayed significantly higher expression of the ChREBP-β isoform. Considering the constitutive activity of this isoform, it suggests a potentially significant role for ChREBP in LUAD.

Functional inactivation of endogenous ChREBP through overexpression of human dnChREBP in the NCI-H1975 LUAD cell line, which exhibited the highest ChREBP expression among the three LUAD lines examined in our study, led to a significant reduction in colony formation and significant alterations in the expression of 650 genes. The number of downregulated genes was approximately 10 times greater than the number of upregulated genes, suggesting that ChREBP primarily functions as a transcriptional activator in these cells. Currently, there is a notable lack of published studies employing loss-of-function approaches to investigate the role of ChREBP in human cancer cells using high-throughput methods (e.g., expression microarrays or RNA sequencing). For comparative analysis, only one study in high glucose-treated HepG2 cells has provided a comprehensive list of ChREBP target genes [12]. That study identified 783 ChREBP target genes using ChIP-seq, with a subset of 129 genes showing greater than two-fold expression increases under high glucose conditions, as revealed by expression microarray analysis. Among these, only 3 genes (*TXNIP, AGT,* and *SLC6A12*) overlapped with our findings, all of which were downregulated in dnChREBP-overexpressing NCI-H1975 cells. Furthermore, within the list of 783 ChREBP target genes identified by ChIP-seq in HepG2 cells, 13 genes (*SYN3, TGM2, DPP4, SLC47A1, NINJ2, TRPM2, PECAM1, PTPRN2, SDK1, CPS1, RORC, PPP1R3C,* and *RFTN2*) were downregulated, and 2 genes (*PDGFRL* and *CYFIP2*) were upregulated in our dnChREBP-overexpressing NCI-H1975 cells. However, the expression levels of these 15 genes in high glucose-treated HepG2 cells were not reported, limiting direct comparisons. Notably, most of established target genes of ChREBP in human cells did not exhibit substantial fold changes in expression in our study. These findings suggest that ChREBP may regulate unique gene networks specific to LUAD cells.

*TXNIP* is a well-documented ChREBP target gene reported in various human cells and tissues, including human pancreatic islets [48], the HepG2 hepatocellular carcinoma cell line [12], and human aortic endothelial cells [49]. Not surprisingly, *TXNIP* is among significantly downregulated DEGs in our experimental findings. TXNIP plays pivotal role in redox homeostasis, glucose and lipid metabolism, cell cycle and inflammation. However, the involvement of TXNIP in cancer is intricate and may have diverse implications depending upon the cancer type and disease stage [50]. Expression of TXNIP mRNA and protein is lower in human lung cancer tissues compared to non-tumor samples [51]. Moreover, TXNIP expression has been implicated in cell viability, proliferation, migration, and invasion in the A549 human LUAD cell model. Notably, lung cancer patients exhibiting elevated TXNIP levels demonstrate diminished progression-free survival rates in contrast to their counterparts with lower TXNIP levels [52]. Our findings regarding ChREBP-mediated regulation of TXNIP expression in LUAD cells identify the ChREBP-TXNIP axis as a potential pathway linking metabolic regulation to tumor growth and progression.

*FASN* is a key lipogenic gene required for proliferation of cancer cells [53]. Previous study have demonstrated that *FASN* silencing induces EMT, promotes metastatic capacity in vitro, and enhances A549 NSCLC cell lung colonization and lethality in vivo [42]. It has been reported that ChREBP and *FASN* are coordinately downregulated in response to TGFβ1 in A549 NSCLC cells. However, in our study, dnChREBP overexpression in NCI-H1975 cells resulted in a significant increase in *FASN* expression, although with log2 FC < 2. This modest increase in *FASN* expression in dnChREBP-overexpressing LUAD cells likely represents an adaptive response to maintain essential lipogenic flux. This finding suggests the activation of compensatory regulatory mechanisms possibly involving SREBP-1, which plays a critical role in regulating *FASN* expression and lipogenesis in human LUAD cells [54,55].

Our differential expressed gene network analysis highlights the enrichment in mechanisms related to gene expression and regulation (transcription coregulator binding and negative regulation of transcription by RNA polymerase II GO pathways), signal transduction (regulation of small GTPase mediated signal transduction, regulation of ERK2 and ERK2 cascade, protein tyrosine kinase activity, protein autophosphorylation, peptidyl-tyrosine phosphorylation, and FGF binding GO pathways), proliferative and survival capabilities of cancer cells (positive regulation of NF-κB transcription factor activity and positive regulation of cell population proliferation GO pathways; TNF-alpha signalling via NF-κB and inflammatory response hallmark pathways), and cell migration, invasion and extracellular interactions (EMT hallmark pathway and extracellular space GO pathway). These results support the reduced colony-forming capability in dnChREBP-overexpressing NCI-H1975 cells, as the altered pathways are closely linked to cell proliferation, survival, and migration-key processes underlying colony formation. This is consistent with a previous study showing that ChREBP suppression by siRNA transfection resulted in diminished proliferative and tumorigenic potential in the HCT116 colorectal cancer cell line [45].

EMT is a process that contributes to cell migration, invasion, tumor progression, metastasis, and drug resistance in cancers, including NSCLC [56]. Our study in dnChREBP-overexpressing NCI-H1975 cells reveals the significant role of ChREBP in regulating the expression of EMT-related genes, providing new detailed insights into its potential involvement in LUAD progression. We observed the downregulation of *FBLN2, IL32, ITGB3, LOX, MMP1, NNMT, PTX3, RGS4, SPP1, TAGLN*, and *VIM*, all of which have been previously reported to be upregulated in LUAD cell lines or tissues from LUAD patients [57–74]. Interestingly, among the 17 hub genes identified in our analysis, 8 are directly associated with EMT, including *BDNF*, *CCN2*, *CXCL1*, *COL1A2*, *COL6A3*, *PDGFRB*, *LOX*, and *SPP1*. In contrast, *CADM1* was the only EMT-related gene found to be upregulated in our experiment. Previous studies have shown that CADM1 expression is reduced in A549 cells, and restoration of CADM1 to normal or higher levels suppresses tumor formation by these cells in nude mice [75]. These preliminary DEGs results suggest that ChREBP may act as a pivotal regulator of the EMT process and underscore the complex interplay of EMT-related genes in LUAD. Consistent with these transcriptomic findings, our transwell migration assay demonstrated a marked reduction in the migratory capacity of dnChREBP-overexpressing NCI-H1975 cells compared with control cells (Fig 2F and G). This observation aligns with a previous report in hepatocellular carcinoma cells, where siRNA-mediated ChREBP knockdown similarly inhibited migration in Hep3B and Huh7 cells [76]. These findings collectively provide functional evidence supporting ChREBP#39;s role in regulating cellular migration capacity, consistent with its potential involvement in metastatic progression across different cancer types.

Our survival analysis using the Kaplan-Meier plotter revealed significant associations between ChREBP expression levels and OS in LUAD patients. Patients with high ChREBP expression exhibited a shorter median OS, which aligns with our experimental findings that dnChREBP overexpression reduces proliferative capacity in NCI-H1975 cells. Furthermore, significant associations with OS were also observed for 4 of the top 10 downregulated genes, 5 of the top 10 upregulated genes, and 5 of the 17 hub genes identified in NCI-H1975 cells overexpressing dnChREBP. Importantly, these genes were not randomly selected but represent the most significantly altered targets and key network regulators of ChREBP identified in our study, thereby strengthening the biological relevance of their prognostic value. It is noteworthy that the direction of correlation between gene expression and patient survival does not always align with the gene#39;s response to dnChREBP in our cell model. This apparent discrepancy likely reflects the complex nature of tumor biology in patients, where gene function can be context-dependent and influenced by the tumor microenvironment, compensatory mechanisms, and post-transcriptional regulation that are not captured in cell line models. These findings suggest that ChREBP may serve as a prognostic marker in LUAD and that ChREBP and its associated genes may collectively contribute to LUAD progression.

While our findings provide valuable insights into the gene regulatory networks influenced by ChREBP, it is important to acknowledge the limitations of our study. First, dnChREBP overexpression was conducted exclusively in the NCI-H1975 cell line, which may not fully capture the diverse mechanisms present in other LUAD cells with different genetic backgrounds. While the preliminary results of dnChREBP overexpression in NCI-H460 cells showed consistent downregulation of 4 of the top 5 target genes (*QPRT*, *COL1A2*, *KLHL4*, *RGS4*; S2 Fig), further validation in additional LUAD cells may reveal important context-dependent regulatory roles of ChREBP. Second, our dnChREBP retains the bHLH/ZIP dimerization motif but lacks a functional transactivation domain, allowing it to heterodimerize with MLX and form transcriptionally inactive complexes that compete with both endogenous ChREBP isoforms. Consequently, dnChREBP interferes with the binding of both the α and β isoforms of endogenous ChREBP to target gene promoters, preventing us from distinguishing their individual roles in gene regulation. dnChREBP interferes with the binding of both the α and β isoforms of endogenous ChREBP to target gene promoters, preventing us from differentiating their individual roles in gene regulation. Third, significant genes with |log2 FC| < 2, which may play important roles in ChREBP#39;s regulatory networks, could have been excluded from our analysis. Fourth, we employed a dominant-negative approach, which inhibits ChREBP activity. This mechanism differs from genetic depletion, which may reveal additional or distinct ChREBP functions. Complementary studies using ChREBP knockdown would therefore help validate the findings from our dnChREBP model.

In summary, our study demonstrates the presence of both ChREBP isoforms in LUAD cells and highlights the potential importance of ChREBP in regulating genes and pathways that may influence tumor growth, metastasis and its potential role as a therapeutic target in LUAD. Future ChIP assays will help verify whether ChREBP directly binds to the promoters of the identified target genes. Additionally, exploring how ChREBP activity influences responses to targeted therapies could enhance the clinical relevance of our findings. Further research, including functional validation and detailed mechanistic studies across a broader range of LUAD cells, is necessary to fully elucidate the extent and significance of ChREBP on biological processes such as EMT and its broader implications in LUAD progression.

## Supporting information

S1 Table Sequences of the primers for RT-qPCR assays used in this study. All primers are listed in the 5′ to 3′ orientation. (XLS)

S2 Table 650 significantly upregulated and downregulated genes (adjusted p-value <0.05) in dnChREBP-overexpressing NCI-H1975 cells with log2 FC > 2. (XLS)

S3 Table Significantly enriched GO terms and Hallmark gene sets. (XLS)

S4 Table Multiple testing correction of overall survival associations for 37 ChREBP-associated genes. FDR-adjusted p-values were calculated using the Benjamini-Hochberg procedure based on p-values from KMplot. The analysis included 37 significant genes identified from the transcriptomic profiling of dnChREBP-overexpressing NCI-H1975 cells, comprising the top 10 upregulated genes, top 10 downregulated genes, and 17 identified hub genes.
(XLS)

S1 Fig Analysis of short-term viability and growth signaling in dnChREBP-expressing NCI-H1975 cells. (A) Cell viability measured by MTS assay at 24, 48, and 72 hours post-doxycycline induction. Data are presented as mean ± SD (n = 3 independent experiments). (B) Western blot analysis of pERK and β-actin protein at 72 hours. (C) Western blot analysis of pAKT and β-actin protein at 72 hours. For (B) and (C), representative blots from three independent.
(PNG)

S2 Fig Validation of top 5 genes downregulated by dnChREBP in NCI-H460 LUAD cells. Relative mRNA expression levels of the top 5 downregulated genes (QPRT, COL8A1, COL1A2, KLHL4, and RGS4) were measured by qRT-PCR in NCI-H460 cells expressing either dnChREBP or control vector. Data are presented as mean ± SD. Asterisks (*) indicate a significance level of p < 0.05 compared to the expression levels in the control cells.
(JPG)
