# Supplementary figures and images for: Unraveling the role of ChREBP in lung adenocarcinoma: Expression, regulatory networks, and potential functional impact
Source: PLoS One. 2026 Apr 30;21(4):e0347907. doi: 10.1371/journal.pone.0347907 (PMC13132224; doi:10.1371/journal.pone.0347907)

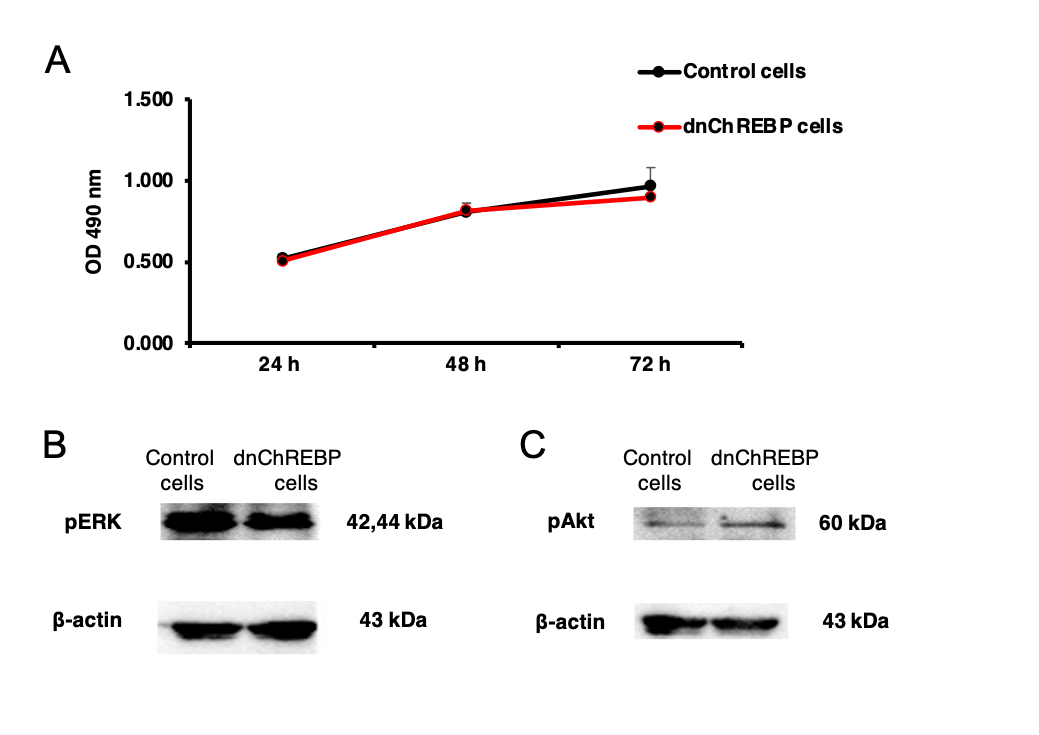

Supplement: S1 Fig — (A) Cell viability measured by MTS assay at 24, 48, and 72 hours post-doxycycline induction. Data are presented as mean ± SD (n = 3 independent experiments). (B) Western blot analysis of pERK and β-actin protein at 72 hours. (C) Western blot analysis of pAKT and β-actin protein at 72 hours. For (B) and (C), representative blots from three independent. (PNG) [file pone.0347907.s005.png]

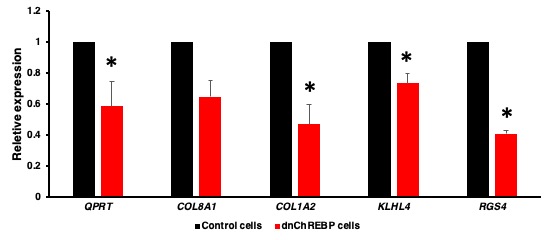

Supplement: S2 Fig — Relative mRNA expression levels of the top 5 downregulated genes (QPRT, COL8A1, COL1A2, KLHL4, and RGS4) were measured by qRT-PCR in NCI-H460 cells expressing either dnChREBP or control vector. Data are presented as mean ± SD. Asterisks (*) indicate a significance level of p < 0.05 compared to the expression levels in the control cells. (JPG) [file pone.0347907.s006.jpg]
